# Attachment to mothers and fathers during middle childhood: an evidence from Polish sample

**DOI:** 10.1186/s40359-019-0361-5

**Published:** 2019-12-11

**Authors:** Anna Kamza

**Affiliations:** SWPS University of Social Sciences and Humanities, Faculty of Psychology and Law, ul. Gen. Tadeusza Kutrzeby 10, 61-719 Poznań, Poland

**Keywords:** Attachment, Security, Avoidant coping, Preoccupied coping, Middle childhood

## Abstract

**Background:**

Middle childhood is a significant period of change both for a child’s cognition and social functioning. Considering that the primary developmental theme of attachment in middle childhood is the balance between child’s growing autonomy and the constant need of relatedness, cultural differences in developmental trends in the attachment might be considered as a function of individualism and collectivism orientations. However, little is known about whether the findings on predictors of individual differences in the attachment in middle childhood found in Western cultures, hold within the non-Western ones. Moreover, still little is known about differences between attachment to mothers and fathers in middle childhood. Hence, one goal of the present study was to investigate the role of a child’s age, sex, and emotionality in a middle-childhood attachment to mothers and fathers in the Polish sample. The second aim was to compare obtained results to the attachment research that focused on Western cultures.

**Methods:**

The sample consisted of 132 children aged 8–12 years (51% boys). They completed the Kern’s Security Scale and the Coping Strategies Questionnaire. Mothers completed a child’s EAS-C and short sociodemographic questionnaire. Pearson’s correlations were conducted to test relationships between a child’s age, sex, emotionality, SES, and attachment-related variables. A paired-samples t-test was used to compare the intensity of preoccupied and avoidant coping strategies with parents in the whole sample. The effects of a child’s age, sex, temperament, and attachment figure were tested with separate repeated-measures ANOVA.

**Results:**

Some of the results replicated prior studies conducted in Western cultures. Similarly to the individualistic cultures, older Polish children reported less preoccupied and more avoidant coping strategies with their parents than younger children. Second, older girls reported higher felt-security with their fathers than with mothers, which suggests some significant changes in attachment relationships regarding the child’s sex. However, as opposed to Western cultures, there were no links between the child’s sex and preoccupied and avoidant coping. Polish children also reported higher rates of preoccupied coping than the avoidant one. Finally, children with relatively lower emotionality reported higher attachment security with both parents than children with relatively higher emotionality.

**Conclusions:**

The current study extends previous work on attachment in middle childhood, the area of rather sparse research, as compared to other developmental periods. The findings reveal the existence of both some specificity in the middle-child attachment in the Polish sample, as well as some culture-universal developmental trends. However, as many questions remain unanswered, they also highlight the strong need for future cross-cultural and comparative studies.

## Background

Middle childhood represents a significant period of change both for a child’s cognition and social functioning. As children become more autonomous and self-reliant, they begin to spend more time away from their parents and start to expand their social networks. They also assume greater responsibility for their behavior [1]. Further significant changes in emotional and cognitive functioning emerge that are also employed in the service of attachment processes. As children begin to develop the capacity for abstract reasoning, as well as cognitive flexibility, they become to employ alternative plans of action [2] better. Development of memory and meta-cognition lead children to better understand different points of view, more effectively regulate their emotions, clearly communicate about them, and to take care of themselves [3]. All those changes manifest in a more proactive approach in a child’s negotiations with the attachment figure and coordinating according to his or her plans with those of the caregiver [4]. They also impact the internal working model of attachment; therefore, studies on attachment in middle childhood are pertinent. Indeed, in recent years, there has been an acceleration of research on attachment in middle childhood; however, many questions remain unanswered. One such question concerns the universality of normative trends in the attachment in middle childhood, related to a child’s essential individual characteristics such as child’s age and sex, that are observed in studies conducted almost exclusively in highly individualistic Western cultures. However, as it will be discussed below, the development of attachment is embedded in particular cultural contexts [4], and thus cultural orientations concerning autonomy and relatedness might influence the development of attachment, especially in middle childhood, when significant individuation-related processes begin. The present paper provides some insight into developmental trends in the attachment in middle childhood by investigating the role of a child’s age, sex, and emotionality on attachment to mothers and fathers in a sample from Polish culture, in which boundaries between collectivistic and individualistic orientations are somewhat blurred.

### Child-parent attachment

Bowlby defined attachment as the emotional bond between an infant and his caregiver, expressing in attachment behaviors (e.g., smiling, vocalizing, crying, and following), the main goal of which is to establish and maintain proximity with the caregiver. The behavioral attachment system is mainly activated by psychological or psychical threat and serves to protect the baby. Currently, it is claimed that the attachment relationship is rather dyad-specific [5]; hence, attachment with the mother may be different from the one with the father or another caregiver. The most important determinant of the child-parent attachment quality is the maternal sensitivity, defined as the caregiver’s ability to accurately perceive and infer the meaning of the child’s signals, and to respond to them instantly and appropriately [6]. The link between maternal sensitivity and attachment security is widely supported by studies in the US and other Western countries [7]. According to the attachment theory [8], the attachment and exploration systems are inextricably linked - children explore their environment when they feel protected and comforted by their caregiver (the so-called “secure base” phenomenon). However, when stressed, children give up their exploratory activities and seek proximity with their attachment figure (the so-called “safe haven” phenomenon). Children who receive responding and calming caregiving and perceive their caregiver as helpful and available, become securely attached. However, when the caregiver is unable to fulfill the secure base and secure haven functions adequately, the child’s sense of security becomes compromised. Two distinct styles of coping with attachment insecurity were identified [9]. The first one, preoccupied attachment, is characterized by a strong need for the caregiver in stressful and novel situations and difficulty in deriving comfort from the caregiver, which results in limitation of the child’s exploratory behavior. On the other hand, avoidant attachment ich characterized by limited affective engagement with the caregiver, avoidance of the caregiver both during exploration and reunion, and failure to seek the caregiver for assistance with coping [6, 9]. The existing evidence suggests that more secure children are more socially and emotionally competent, as compared to insecure children [10], and that the attachment patterns are quite stable over time [11].

### Developmental trends in attachment in middle childhood

In attachment literature, middle childhood is characterized as a time when changes in the intensity of attachment behaviors and conditions activating and terminating the attachment system occur. According to Mayseless [12 p14], a decrease in the intensity of attachment behavior in middle childhood is impacted by “preparations for refocusing and reorienting the investment in affectional attachment bond between children and their parents or primary caregivers to others and their autonomy.” Nevertheless, it is claimed that children in middle childhood continue to use their parents as secure bases supporting exploration and secure havens in a time of stress; thus, parents remain the principal attachment figures. Due to a growth in self-regulation skills in middle childhood, the goal of the attachment system changes from *proximity* to the attachment figure (as in early childhood) to the *availability* of the attachment figure [8]. The latter one is reflected in open communication between parent and child, parental responsiveness to child needs, and the parent’s physical accessibility to the child [13]. However, in the attachment research, there was relatively little attention to the child’s characteristics underlying individual differences in the attachment in middle childhood, such as the child’s age, sex, or temperament. Moreover, still only few studies include fathers as attachment figures, thus still little is known about the differences between attachment to mothers and fathers in middle childhood.

Among existing studies, Lieberman, Doyle, and Markiewicz [14] observed some significant changes in attachment security during middle childhood; 12–14 year-olds reported less relying on mothers and fathers than did 9–11 year-olds, however, children’s perceptions of parents’ availability did not vary with age. Moreover, it was found that preoccupied coping with respect both to mother and father declined with age, but avoidant coping inclined [1, 9]. These results suggest that age changes in attachment styles in middle childhood ought to be interpreted within the context of children’s increasing independence, autonomy from parents, and decision-making [1].

A growing body of evidence also suggests that some sex-specific aspects of attachment styles emerge in middle childhood [e.g., 15]. Namely, girls are classified more frequently as secure or ambivalent while boys - as avoidant or disorganized, and those trends are observed both in normative and different risk samples [e.g., 16] and hold across different assessment methods [9, 17, 18]. It is worth to note that in some studies on adult attachment, similar patterns were found, and it is observed cross-culturally [19]. Based on previous studies, it seems that those results are not likely to be measurement-specific or attributable to cognitive and language development. Del Giudice [15, 20] argue that the emergence of sex differences at around 8-years old is related to a reorganization of the endocrine mechanisms (*adrenarche*) that impact brain development, and thus triggers sex-specific psychological trajectories, which are supposed to be part of a broader shift towards sex-specific psychosocial reproductive strategies in early adulthood. Girls display more ambivalence (preoccupation) to maximize relatedness and support from the family. Boys, on the other hand, display more avoidance and emotional distance, accompanied by autonomy, competition, and status-seeking in the same-sex peer group [15]. An alternative explanation emphasizes social influences on the development of attachment; in the course of socialization, girls are taught to show affiliate responses to regulate negative feelings when stressed, while boys are spurred to react in a *fight-or-flight* fashion [21].

In middle childhood, some diversification in forming affectional bonds with mothers and fathers occurs, and different conditions that activate the attachment system leading a child to look for support and protection from different attachment figures [22]. Mothers are typically seen as the secure havens to whom children turn in the case of distress, hurt, or sickness. Fathers, in turn, are thought to be likely to serve more as secure bases and playmates who expose children to challenging games and activities [23]. However, research findings are mixed, with some studies showing increasing paternal availability over time [1], other reporting lower felt security with mother than with father [24]. Some results also indicate that fathers’ involvement with their children increases as their children grow older, while mothers’ involvement is rather constant [25]. However, the studies mentioned above were conducted in Western cultures (mainly in the U.S. and Canada); therefore, it is challenging to state whether results would be similar in different than Western societies. One could expect somewhat different patterns of those trends due to the differences in fathering views and practices that are products and expressions of culture [26].

Moreover, the interaction of a child’s and parent’s sex may be one of the crucial factors in children’s attachment during middle childhood. Some evidence exists that fathers tend to be more involved with their sons than with daughters, since fathers and sons may identify with one another more and share similar interaction styles [27]. The attachment research seems to confirm those results; in the study of Diener and colleagues [28], girls reported significantly higher attachment security with their mothers than with their fathers, and boys reported significantly higher attachment security with their fathers than did girls. Western studies also reveal some specificity in links between attachment figure and the type of attachment insecurity in middle childhood. In Boldt, Kochanska, Grekin, and Brock’s study [29], child attachment avoidance was higher with fathers, but ambivalence and disorganization - with mothers. Those results might reflect that children probably tend to be more restrained with fathers and more expressive with mothers, which results from differences in parental responsiveness to children’s emotional cues. Some evidence suggests that in Western cultures, fathers use more punitive emotion socialization strategies than mothers do [30]. However, those findings have not yet been replicated in other cultures; thus, it is difficult to say whether the differences in attachment security with mothers and fathers among boys and girls are culture-universal or emic.

Relatively less is known about other than age and sex child’s characteristics related to individual differences in middle childhood attachment. Meanwhile, it should be noted that compared to earlier developmental periods, children in middle childhood undergo more influences outside the family and are more able to shape their environments and social interactions on their own, accordingly with their preferences and innate predispositions [1]. Thus, Bosmans and Kerns [4] argue that in middle childhood (as compared to infancy), parent-child relationships might be more shaped by the dynamics of gene-environment interactions, with even more extensive effects of biologically determined factors on attachment. One such factor might be the child’s temperament, an innate and heritable set of traits that remain stable over time [31]. Temperament, as a biologically determined basis of personality, seems to be one of the most malleable factors underlying individual differences in middle childhood attachment, as it determines a child’s emotional reactivity, as well as the way people relate to each other [31]. However, thus far, research has mainly focused on the role of temperament in the early attachment [for a review, see: 32], whereas less attention has been given to the links between temperament and attachment in middle childhood, although it is widely recognized that the quality of child’s attachment is a product of the interaction between the child’s biological dispositions and the quality of parental care [32]. Since traits such as a child’s sensitivity to stimuli causing distress and a tendency to experience fear, anger, and dissatisfaction [31] play a crucial role in emotion regulation and self-regulatory processes, those dispositions seem to be valid in the context of parent-child interactions [33].

On the other hand, also attachment styles are closely related to emotion regulation strategies, as a child employs those styles in an attempt to get basic attachment needs meet accordingly to the attachment figure’s responsiveness [34]. However, in opposite to temperament, attachment is not inherent, but instead, a child rebuilds attachment representations through the interactions with the primary caregiver [35]. Bowlby [8] argued that a child not only integrates new experiences into existing internal working models of attachment (assimilation) but also revises them to accommodate current experiences with an attachment figure (accommodation). Admittedly, one of the core tenets of attachment theory states that the quality of the child-parent attachment depends at most on the caregiver’s sensitivity and availability to the child’s cues, and his response is learned in the interaction with the caregiver and set in internal working models. However, it was observed that in middle childhood, children who are more emotionally reactive tended to be more vulnerable to experience distress and interpreted mother’s ambiguous behaviour as unsupportive, regardless of the objective meaning of her behaviour [36]. Hence, the concern arises that as children grow older and their thinking becomes more abstract and reflective, those with high negative emotionality might relatively more intensively assimilate such biased interpretations in their attachment representations, and they might use specific secondary attachment strategies more profoundly than children with low negative emotionality. Some research has shown that children who have high levels of difficult temperament were less capable of utilizing their attachment representations to regulate their emotions [e.g., 35]. However, there is a lack of research concerning emotionality in the context of normative trends in the attachment in middle childhood, and no research investigated its potential interactions with age, sex, and attachment to parents in that developmental period.

### Attachment in the context of culture

Although those relatively small number of current findings add substantially to the knowledge about attachment in middle childhood, one of the major problems is that most of the studies have been primarily confined to Western contexts. Surprisingly, little is known about whether the findings on predictors of individual differences and development in the attachment in middle childhood found in Western cultures, hold within non-Western ones. Meanwhile, the development of attachment is embedded in particular cultural contexts of socio-political, historical, and economic circumstances [4]. As Keller [37 p189] points, “independence from others and personal autonomy are the ideological foundations of attachment theory with notable consequences for the definition of parenting quality, childrearing goals, and with respect to an understanding of desirable endpoints of development.” Indeed, cultures differ significantly in their models of autonomy and relatedness and related to them childrearing practices or parent-child behavioural relationships [38]. Considering that the central developmental theme of attachment in middle childhood is the balance between a child’s growing autonomy and the need for relatedness, cultural differences in developmental trends in the attachment might be considered in terms of individualism and collectivism orientations [39]. Within individualistic cultural contexts (e.g., the U.S. or Western Europe), people place relatively greater emphasis on independence and autonomy. In contrast, within collectivistic cultural contexts (e.g., Japan or China), people place a higher weight on interdependence and relational harmony [40].

Indeed, individualistic and collectivistic values may impact the development of the behavioural attachment system [38], but there is a lack of empirical studies systematically testing the cross-cultural differences in developmental trends in middle childhood attachment, and the factors explaining it. Meanwhile, recent evidence suggests that cultural differences in attachment go far beyond the differences in the distribution of the attachment styles [37, 41]. For instance, Mizuta and colleagues [42] found that Japanese and US dyads did not differ in attachment security and maternal sensitivity during separation-reunion episodes, but Japanese preschoolers showed more need for bodily closeness (*amae*) than US preschoolers. Moreover, *amae* was positively linked to internalizing behaviours in US children but not for Japanese ones, which suggests that *amae* can be one of the culture-specific attachment-related behaviours. Other comparisons of the U.S. and Japan studies also reveal the cultural relativity of three core hypotheses of attachment theory: that maternal sensitivity is the antecedent of secure attachment, that secure attachment leads to social competence, and that securely attached children use the caregiver as a secure base for exploration [7]. For example, the primary function of maternal sensitivity in an individualistic view is to foster a child’s exploration and autonomy, assert his or her desires, and to promote the child’s individuation [7]. By contrast, in collectivistic cultures, mothers labelled as sensitive are expected to react in anticipation of children’s signals, and their reactions promote a child’s relatedness and emotional closeness. Here the primary function of sensitivity is to help the child regulate his or her emotional states and to promote the child’s social engagement and interdependence [7, 43]. Such different notions about the functions of maternal sensitivity are also linked with the way attachment theorists define social competence. In individualistic cultures, this competence entails mainly exploration, autonomy, and a positive view of self [7], which is essential for self-dependence. In opposite, in the collectivistic culture of Japan social competence often means dependence, self-criticism, and the ability to coordinate one’s needs with the needs of others [7]. There is also some evidence that even the link between attachment and exploration seems to be less primary in non-Western cultures [37], where attachment security is more strongly linked to social dependence and loyalty. At the same time, in Western societies, strong relations between attachment security, individuation, and autonomous mastery of the environment are consequently observed [37]. On the other hand, as Bakermans-Kranenburg and collaborators [44] postulate, in attachment research, the role of culture should not be confused with the impacts of socioeconomic status (SES). In their study, those authors found that even though there was a similar correlation pattern between maternal sensitivity and infant attachment security, African-American children scored lower on attachment security than the white children. Further analyses revealed that African-American ethnicity was related to lower-income, which in turn affected infant-mother attachment.

### What about Poland?

Despite the growing recognition that in the current era of globalization and socio-political changes individualist–collectivist depictions of value systems and developmental goals are overly simplistic [38], little (if any) is known about the specificity of attachment in the so-called *cultures of social change* [45], as those studies instead focus on the Eastern-Western dichotomy. In those cultures, which are typical for most post-communist countries, the boundaries between collectivistic and individualistic orientations are somewhat blurred. Despite the rapid institutional changes, there is a much slower change in social values, and simultaneous socialization of dependence and independence occurs [46]. Such an example might be fostering independence in children, which is thought to lead to the enhancement of relational skills [38].

An example of such a culture of social change is the Republic of Poland, an ethnically homogenous country located in Central Europe, which in the last three decades, has undergone a swift transition to capitalism and democracy [47]. At the end of June 2017, the population of Poland amounted to 38 million people, with 6.9 million children aged 0–18 (35% of which were in middle childhood [48]). However, there are relatively few studies on child-parent attachment in Poland. For example, the study of Czyżowska and Gurba [49] confirmed the general hypothesis about the impact of child-mother on the later adult relationship with romantic partner: closeness experienced in relationships with parents during childhood and adolescence was related to the feeling of intimacy with one’s partner which in turn had an impact on the perceived quality of the relationship. Another Polish study [50] revealed that adolescents suffering from mixed disorders of conduct and emotions perceived their parents as less protective and revealed a higher level of anxiety than did the control group. However, to the best of the author’s knowledge, there is a lack of Polish studies on predictors of individual differences in middle childhood attachment. Moreover, still little is known about the differences between attachment to mothers and fathers. Therefore, it is difficult to say whether the findings from other cultures hold within the Polish samples.

In Poland, the most of traditional parenting practices still promote connection to the family and other close relationships, respect and obedience [51], but at the same time Polish parents believe about the fundamental requirements for children’s achievement of autonomy, personal choice, intrinsic motivation, and self-esteem [46]. Trommsdorff and Nauck [52], in their Value of Children study found, that in Poland, there is greater valuing of such developmental goals as obedience in the family and popularity among other people, comparing to Germany, which is seen as a highly individualistic society. In turn, Hofstede [53] points to a smaller individualistic orientation in Poland than in Germany and in other Western Europe countries. Another study [46] revealed that Polish mothers are more collectivistic in their socialization goals than German mothers, and also their parenting practices are more in line with those values. Moreover, Lubiewska [46] pointed out that due to the fast cultural changes in Poland in the last decades, there exist micro-cultural discrepancies between relatedness-oriented mothers and their autonomy-oriented children, what creates an interesting question about developmental trends in the attachment in the period, when children expand their social worlds and gain more autonomy. At the same time, Kerns and colleagues [1] claim that depending on social values (e.g., independence vs. interdependence) in different cultural contexts, the decline in utilization of parents may emerge at different times. However, to the best of the author’s knowledge, no systematic research on developmental trends in the attachment in middle childhood was conducted in Poland. Hence it is difficult to compare those trends to another culture, especially in the context of coexistence of autonomy-relatedness values.

### The current study

As it has been mentioned before, relatively little is known whether the findings on predictors of individual differences in the attachment in middle childhood found in Western cultures hold within the non-Western ones. Moreover, relatively little attention is paid to the child’ s characteristics underlying individual differences in the attachment with fathers as compared to attachment with mothers in middle childhood. Therefore, the first purpose of this study was to examine the role of a child’s age, sex, and emotionality in a middle-childhood attachment with both parents in the Polish sample. The second aim was to compare the obtained results to those focused on Western cultures.

The recent results show that in Poland, most of the traditional parenting practices still promote relatedness, respect, and obedience [51], and the Polish mothers are still rather collectivistic in their socialization goals. There is also a higher valuing of obedience in the family and popularity among other people, compared to other Western Europe countries [53]. Therefore it was predicted that in general Polish sample, children would report more preoccupied than avoidant coping strategies with their parents (hypothesis 1).

Furthermore, in middle childhood, specific components of the attachment relationship may remain stable with age, while others may change [14]. Moreover, in different cultural contexts, the decline in the utilization of parents may emerge at different times, depending on social values (e.g., independence vs. interdependence [1]). Therefore, it was expected that older children would report more avoidant coping strategies with their parents than younger children (hypothesis 2), but there would be no age differences in preoccupied coping strategies (hypothesis 3).

Beyond the proposed culture-specific hypotheses*,* a culture-universal link between a child’s sex and attachment insecurity was also hypothesized. Existing findings reveal the existence of universal, biologically-based reorganization of the endocrine mechanisms triggering sex-specific psychological trajectories in middle childhood [15, 20]. There is also cross-culturally observed specificity in gender-socialization practices in which girls are taught to show more affiliate responses than boys [21]. Hence, it was expected that girls would report more preoccupied coping strategies with their parents than boys (hypothesis 4), and boys would report more avoidant coping strategies than girls (hypothesis 5).

Another aim of the present study was to test the role of emotionality (a temperamental trait depicting the negative emotionality and intensity of emotional reactions) in middle childhood attachment. It was observed that in middle childhood, children who are more emotionally reactive tended to be more vulnerable to experience distress, and learn to interpret the mother’s ambiguous behavior as unsupportive [36]. Given that biologically determined factors might have more substantial effects on attachment than during infancy [4], it was expected that emotionality would be positively linked to avoidant (hypothesis 6a) and preoccupied (hypothesis 6b) coping, and negatively to attachment security (hypothesis 6c) only in older children.

Regarding the fact that research is unclear to allow one to relate a child’s sex and age to attachment security and coping strategies in an emotionality-specific way, the moderating role of temperament in those links was tested as an exploratory part of this study.

The present study also had one more goal. Namely, still unexplored are the differences between mother-child and father-child attachment in middle childhood, and this fact applies both to Western and non-Western cultures. As it has been said previously, some authors suggest that mothers are typically seen as the secure havens, and fathers tend to serve more as secure bases [23]. Research findings are mixed, with some studies showing increasing paternal availability over time, as fathers’ involvement with their children increases as their children grow older [1]. There is also scarcity in studies on fathering in Poland. Therefore, given a lack of a strong theoretical rationale, the effects of the parental figure on a child’s security, preoccupied, and avoidant coping was also tested as another exploratory part of this study. Considering the role of a child’s age, sex, and temperament, and how the attachment representations regarding mother and father may vary from one another may help us to better understand each parent’s unique contribution to attachment development in middle childhood.

Understanding the developmental trends in the attachment to mothers and fathers, as well as the roles of child characteristics and gender in middle childhood, represent essential questions in developmental research. Comparing results of this study to the bulk of attachment research that focuses on Western cultures would enrich our knowledge not only about the developmental trends and individual differences in middle-childhood attachment but also it could help to understand the role of culture in that phenomenon. Finally, examining the role of the parent’s sex and child’s emotionality in attachment might help to better understand the underpinnings of individual differences in the attachment in middle childhood.

## Methods/design

### Participants

The present study was a part of a larger research project investigating relations between attachment, executive functions, and mentalization in middle childhood, directed by the author of the present paper. Participants were recruited through seven public elementary schools in a large metropolitan area in Poland. The schools were selected randomly and should not differ systematically from other mainstream schools from larger Polish agglomerations. Letters were sent to parents of children explaining the nature of the study, and informed parental consent was obtained for the participants. The initial sample consisted of 165 children. However, 26 questionnaires from mothers did not return, and 6 of them were incomplete. Therefore, the main analyses were conducted on data from 132 children (51% boys) aged 8–12 years (*M* = 9.97 years, *SD* = 1.41 years; for sample summary – see Table 1). The number of children in particular age groups did not differ significantly, χ2(4) = 0.06, *p* = .99. The number of boys and girls in particular age groups can be considered as not significantly different: χ2(1) = 0.19, *p* = .73 for 8-year-olds; χ2(1) = 0.27, *p* = .60 for 9-year-olds; χ2(1) = 0.03, *p* = .86 for 10-year-olds; χ2(1) = 0.27, *p* = .60 for 11-year-olds and χ2(1) = 0.12, *p* = .72 for 12-year-olds. The sample was White European, quite homogeneous in terms of SES (middle-class families; see also the Results section), and consisted of two-parent families. The population of Poland is ethnically homogeneous; therefore, the sample selected for the present study can be considered representative.

**Table 1:** Sample Summary Table (N = 132)

Variable		*n*	*M*	*SD*	Range
Age (months)					
	8 year-olds	26	99,79	3,25	96–106
	9 year-olds	29	114,48	2,85	108–119
	10 year-olds	25	123,48	2,55	120–129
	11 year-olds	27	136,35	3,83	132–143
	12 year-olds	25	145,87	3,14	134–152
	total sample	132	123,92	16,66	96–152
SES (composite)		132	8,86	2,39	2,75–11,25
Sex					
boys		67			
girls		65			

## Measures

### Procedure

First, the Ethics Committee for Research Projects at the Institute of Psychology in Adam Mickiewicz University’s approval was obtained for the project. Recruitment for the study was conducted in seven elementary schools and was based on voluntary submissions. Written informed consent was obtained from both the headteacher of each school involved and the parents of all children, before their participation. Families were invited to participate provided that they had a child participant in the age range 8–12, and who were living with both biological parents. Given that attachment styles built on experiences within one’s family of origin during early childhood are fairly stable from infancy to middle childhood [11], non-biological families were excluded in order to provide the relative stability of children’s attachment bonds. Given also the broader context of the present study (as it was mentioned before, the study was a part of a larger research project investigating relations between attachment and cognitive functioning in middle childhood), children with learning disabilities were also excluded. The screening was based on the information from the sociodemographic questionnaire (see the *Measures* section). The positive response rate was 35%.

As the attachment measures were common for child-mother and child-father bonds, the study was carried out in two sessions separated by a one week break, which served as a procedural remedy both to prevent children’s’ fatigue and discouragement, and to control for common method bias [54]. In the first session, children reported on attachment to one parent, whereas in the second session, attachment to another parent was assessed. Children were assessed individually in a quiet room in their schools by a female experimenter. Considering the possible reading difficulties among our young participants, the experimenter read aloud the items from the two attachment questionnaires before the child made the answer selection. After completion of the study, children received small gifts (sweets and stickers). Between the sessions, mothers were asked to complete the EAS-C and the short sociodemographic questionnaires. No data were missing for the variables used in the final sample.

### Child’s perceptions of attachment security

Child attachment security was assessed separately for mother and father using the Polish adaptation [55] of the Kern’s Security Scale (KSS) [56], a 15-item, a self-report measure which is widely used in the United States and certain European countries. The scale assesses the child’s perceptions of the availability and responsivity of the attachment figure, the child’s tendency to rely on the attachment figure in the time of stress, and ease and openness in communicating with the figure. Using Harter’s [57] “Some kids. .. other kids. ..” format, the children were asked to indicate which statement was most like them, and then to indicate whether it was “sort of true” or “really true.” Each item on the Security Scale was scored from 1 to 4 with higher scores indicating a more secure parent-child attachment. Previous research has demonstrated the validity of this measure (see Kerns et al., 1996; Kerns et al., 2005), with good internal consistency (for an overview, see [58]) and high test-retest correlation, *r* (30) = .75, over a short time interval. The scale was also shown to have meaningful associations with other attachment measures and caregiver sensitivity [34, 56, 59]. Furthermore, the Security Scale showed significant associations with developmental correlates of attachment, such as school adaptation, emotional and peer social competence, self-esteem, and behavioural problem [58].

The text of the KSS was translated to the Polish language by an expert psychologist, and then another independent psychologist translated it back to English. Disagreements in translations were resolved through discussions about the meaning of the source items. The final version of the items, supervised by an expert in attachment research, revealed to be satisfactory. Consistent with prior works [60], our CFA results provided evidence for the unidimensional structure of the KSS [55]; (CFA results available upon request). The scores were averaged to produce a single score on the continuous dimension, with a higher score reflecting perceptions of greater attachment security. In the present study, Cronbach’s alphas were .75 and .76 for security with mothers and fathers, respectively.

### Child’s preoccupied and avoidant coping strategies

Younger, Corby and Perry [61] claim that continuous assessment of attachment security allows only the relative positioning of the child on the continuum from the least to the most secure attachment, while it does not say anything about the specificity of the attachment insecurity, what may lead to incomplete conclusions. Therefore, the Coping Strategies Questionnaire (CSQ) [9], in the Polish adaptation by Kamza and Głogowska ([62], was used to assess preoccupied and avoidant dimensions of insecurity with both parents. Like the KSS, the CSQ is a self-report measure and initially consists of 36 items (18 items per scale), which are rated using the Harter’s ‘Some kids … , but other kids … ’ format. Each item describes a situation in which a child is in distress and asks the child to choose which would be his or her most likely response. The Preoccupied Coping Scale assesses the child’s over-dependency, a strong need for the attachment figure under stressful or novel situations, anxiety about separation from the attachment figure, inability to be comforted by the attachment figure in stressful situations, and difficulties in exploring or dealing with challenges owing to excessive need for the figure*.* The Avoidant Coping scale assesses the child’s denial of the need for the attachment figure when stressed, unwillingness to use him or her as an emotional or task-relevant resource. Each item of the Preoccupied and Avoidant Coping scales was scored as 0, 1, or 2, with higher scores reflecting more preoccupied or avoidant coping strategies. The Coping Strategies Questionnaire has been shown to have construct validity in that children’s specific styles of using their caregiver to cope with everyday stressors were related in predictable ways to adjustment and parent-child relationships during middle childhood [9, 61]. The measure showed to have good internal consistency; in Kerns’ study [1], alphas for preoccupied and avoidant coping with mother were .84 and .69, respectively, and for preoccupied and avoidant coping with father were .83 for both.

In the present study, a short version of the Polish version of the Coping Strategies Questionnaire consisting of 20 items (10 for each scale) [13, 63] was used because of time constraints [62]. Items that showed high item-total correlations were selected. In the present study, Cronbach alphas for preoccupied and avoidant coping with mother were .79 and .65, respectively, and for preoccupied and avoidant coping with father were .84 and .66 respectively. CFA results provided evidence of the two-dimensional model of the CSQ fit the data well and achieved a good model fits both for mother-child attachment [62]. Item scores were averaged so that children received total scores on continuous dimensions of preoccupied and avoidant coping.

### Child emotionality

Mothers were asked to complete the five-item child’s Emotionality Scale from the Emotionality, Activity, and Sociability Survey for Children: Parental Ratings (EAS-C) [31] in Polish adaptation by Oniszczenko [63]. The scale measures the negative quality of the child’s emotions and the intensity of emotional reactions. Using a 5-point scale, mothers rated how characteristic each description was of their child (1 = “not characteristic or typical of your child,” 5 — “very characteristic or typical of your child”). The score was computed by summing the five items, with higher scores indicating greater negative emotionality. The scores in the current sample were dichotomized at the median (*Me* = 14.5) to form two groups, relatively high and relatively low in emotionality, which were then compared for their means on the attachment dependent variables (see further analyses). The internal consistency of the emotionality scale was adequate (Cronbach’s α = .73).

### Sociodemographic questionnaire

A short sociodemographic questionnaire was used, concerning essential child’s characteristics (date of birth, sex) and their family socioeconomic status (mother’s and father’s education, their status of employment, and family living area). Additionally, the screening question about whether the family is a child’s biological one was included. The general SES family index was calculated, taking into account different weights for the three components of SES: 1.0 for parents’ education, 0.5 for employment status, and 0.25 for a place of residence. The higher the value of that index, the higher the family SES (range: 2 [i.e., both parents with primary education, not working, living in the countryside] - 11.25 [i.e., both parents with higher education, working, living in the city]).

### Data analyses

One hundred sixty-five children filled the Security Scale and Coping Strategies Questionnaire. However, there were only 132 EAS questionnaires returned completed, hence the further main analyses were conducted with 132 cases. All statistical analyses were performed using Statistical Package for the Social Sciences (SPSS) version 25. All tests were 2-tailed with α = .05. First, distributions of all continuous scores were screened for normality. No multivariate outliers were found, and all of the distributions were within bounds of moderate normality (skewness < 3.0; kurtosis < 7.0) [64], therefore parametric tests were used.

Results of a preliminary set of analyses are reported first; descriptive statistics were calculated to summarize the data, and Pearson’s correlations were conducted to compare relationships between child’s age, sex, emotionality, SES, and the attachment-related variables. Next, a paired-sample t-test was used to compare the intensity of preoccupied and avoidant coping strategies with parents in the whole sample (hypothesis 1).

Due to the complexity of hypotheses and exploration problems, a stricter significance threshold for individual comparisons (and repeated-measurement in the case of attachment to mother and father) was needed to compensate for the number of inferences being made. Therefore, instead of testing each hypothesis separately, the effects of a child’s age, sex, temperament, and attachment figure were tested jointly for each attachment variable with separate repeated measures univariate analyses of variance (ANOVA). This statistical approach should provide greater insight into the connections between the studied variables.

## Results

### Preliminary analyses

Descriptive statistics for attachment variables are shown in Table 2, and for emotionality – in Table 3.

**Table 2:** Means, Standard Deviations, and the Ranges in Scores for Attachment-Based Measures by the Child’s Sex and Age

Sex	Age	*N*	Avoidant Coping				Preoccupied Coping				Security			
			Mother		Father		Mother		Father		Mother		Father	
			*M (SD)*	Range	*M (SD)*	Range	*M (SD)*	Range	*M (SD)*	Range	*M (SD)*	Range	*M (SD)*	Range
Boys	8	12	0.15 (0.20)	0.00–0.50	0.13 (0.11)	0.00–0.30	1.01 (0.50)	0.20–1.80	0.92 (0.59)	0.30–2.00	3.25 (0.47)	2.27–4.00	3.25 (0.270	2.80–3.67
	9	15	0.13 (0.13)	0.00–0.40	0.13 (0.19)	0.00–0.60	0.89 (0.50)	0.10–1.80	0.93 (0.59)	0.30–1.90	3.20 (0.39)	2.40–3.73	3.27 (0.40)	2.27–4.00
	10	13	0.25 (0.21)	0.00–0.60	0.16 (0.28)	0.00–0.90	0.90 (0.64)	0.00–2.00	0.92 (0.67)	0.20–2.00	3.18 (0.38)	2.47–3.87	3.32 (0.42)	2.40–3.80
	11	16	0.25 (0.21)	0.00–0.70	0.29 (0.23)	0.00–0.80	0.67 (0.47)	0.00–1.60	0.51 (0.40)	0.00–1.20	3.20 (0.47)	2.07–3.73	2.95 (0.42)	2.07–4.00
	12	11	0.53 (0.32)	0.00–1.10	0.33 (0.29)	0.00–0.80	0.40 (0.22)	0.10–0.90	0.39 (0.28)	0.00–1.00	3.07 (0.38)	2.00–3.53	3.18 (0.25)	2.80–3.67
Girls	8	14	0.14 (0.11)	0.00–0.30	0.17 (0.26)	0.00–0.80	0.98 (0.46)	0.00–1.80	0.91 (0.50)	0.20–1.80	3.16 (0.29)	2.67–3.67	3.17 (0.38)	2.40–3.73
	9	14	0.20 (0.25)	0.00–0.80	0.14 (0.22)	0.00–0.80	0.81 (0.43)	0.20–1.80	0.73 (0.53)	0.20–1.80	3.23 (0.51)	1.93–4.00	3.15 (0.34)	2.73–3.80
	10	12	0.09 (0.22)	0.00–0.70	0.18 (0.29)	0.00–0.90	1.01 (0.49)	0.20–2.00	1.06 (0.62)	0.00–2.00	3.27 (0.48)	2.33–4.00	3.14 (0.79)	1.53–4.00
	11	11	0.19 (0.30)	0.00–0.90	0.21 (0.21)	0.00–0.60	0.65 (0.38)	0.20–1.50	0.62 (0.30)	0.10–1.10	3.20 (0.44)	2.27–4.00	3.10 (0.38)	2.73–4.00
	12	14	0.26 (0.30)	0.00–1.10	0.28 (0.26)	0.00–0.70	0.43 (0.25)	0.00–0.90	0.41 (0.38)	0.00–1.10	3.17 (0.55)	2.40–3.73	3.41 (0.42)	2.53–3.67
Boys		67	0.24 (0.23)	0.00–1.00	0.21 (0.23)	0.00–0.90	0.77 (0.51)	0.00–2.00	0.74 (0.56)	0.00–2.00	3.19 (0.42)	2.07–4.00	3.18 (0.38)	2.07–4.00
Girls		65	0.18 (0.24)	0.00–1.10	0.20 (0.24)	0.00–0.90	0.79 (0.47)	0.00–2.00	0.74 (0.52)	0.00–2.00	3.24 (0.47)	1.93–4.00	3.19 (0.48)	1.53–4.00
Total		132	0.21 (0.24)	0.00–1.10	0.20 (0.24)	0.00–0.90	0.78 (0.49)	0.00–2.00	0.74 (0.54)	0.00–2.00	3.21 (0.44)	1.93–4.00	3.19 (0.43)	1.53–4.00

**Table 3:** Means, Standard Deviations, and Ranges for Emotionality by the Child’s Age (N = 132)

Age	*N*	*M (SD)*	Range
8	26	14.15 (3.00)	9–20
9	29	15.31 (4.13)	10–25
10	25	15.04 (3.37)	11–22
11	27	15.00 (3.82)	9–25
12	25	14.24 (3.29)	8–20
Total	132	14.77 (3.54)	8–25

Preliminary analyses revealed that there were neither significant main effects of age (*F* (4, 97) = 1.70, *p* = .16, η_p_^2^ = .07) and sex (*F* (1, 97) = 0.04, *p* = .85, η_p_^2^ = .00) on emotionality, nor their interaction (*F* (4, 97) = 1.03, *p* = .39, η_p_^2^ = .04). Furthermore, the sample was quite homogenous in terms of the SES (*M* = 8.86, *SD* = 2,39, range: 2.75–11.25; 80% of mothers and fathers had a master’s level or a professional degree, 90% had a full-part work, and all of them lived in a big city; see also Table 1).

### Associations among age, sex, emotionality, and attachment

Pearson’s zero-order correlation coefficients and the biserial point coefficients (for sex) were calculated to examine associations among child individual characteristics and attachment dimensions. The results are presented in Table 4. Moderate and positive relations between age and avoidant coping with both parents (*r* (130) = .25, *p* < .01) and negative with preoccupied coping with mother (*r* (130) = −.33, *p* < .001) and father (*r* (130) = −.34, *p* < .001) were found. On the other hand, child age was not related to attachment security (*p*s > .05). Emotionality correlated negatively and weakly with attachment security with mother (but not with the father) *r* (130) = −.20, *p* < .05. There were no significant correlations between attachment and sex (all *p*s > .05). Finally, there were no significant associations between family SES and child variables (all *p*s > .05). As such, SES was not included in subsequent analyses.

**Table 4:** Zero-Order Pearson Correlations among Child’s Age, Sex and Attachment-Based Measures (N = 132)

Variable				Mother			Father		
	SES	Age	Sex	Security	Preoccupied Coping	Avoidant Coping	Security	Preoccupied Coping	Avoidant Coping
Sex	–	–	–	−.03	−.03	.14	−.03	−.00	.02
Age	–	–	.01	−.05	−.33***	.25**	−.03	−.34***	.25**
SES	–	.09	.05	.07	.04	−.03	.02	.03	.03
Emotionality	.15	−.00	.00	−.20*	.07	.05	−.10	.05	.09

*p < 0,05; **p < 0,01; *** *p* < 0,001

Although not correlated to the most of attachment measures (all *p*s > .05, except the aforementioned link between emotionality and attachment security with mother), child’s sex and emotionality were included in subsequent ANOVA analysis (see next sections) due to the fact that they might serve as potential moderators of the links between child characteristics or attachment figure and the dimensions of child attachment.

### Preoccupied and avoidance coping strategies in the general sample

**Fig. 1:**
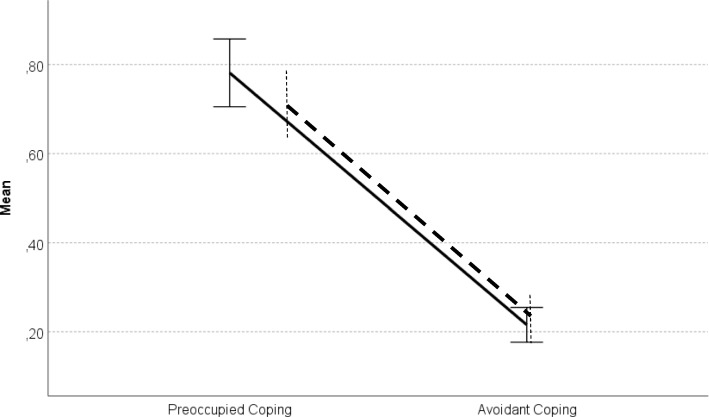
Preoccupied and avoidant coping with mothers and fathers in the whole sample. *Legend*: error bars represent the standard error; solid line – attachment to mother; dashed line – attachment to father

To check whether, in the general Polish sample, children would report more preoccupied than avoidant coping strategies with their parents (hypothesis 1), we used the paired-sample *t*-test. It revealed that children reported higher preoccupied coping both with mothers (*t* (164) = 11.62, *p* < .001, Cohen’s *d* = 1.48) and fathers (*t* (164) = 9.67, *p* < .001, Cohen’s *d* = 1.32) than the avoidant coping (see Table 2 and Fig. 1).

### Age, sex and emotionality differences in avoidant coping

**Fig. 2:**
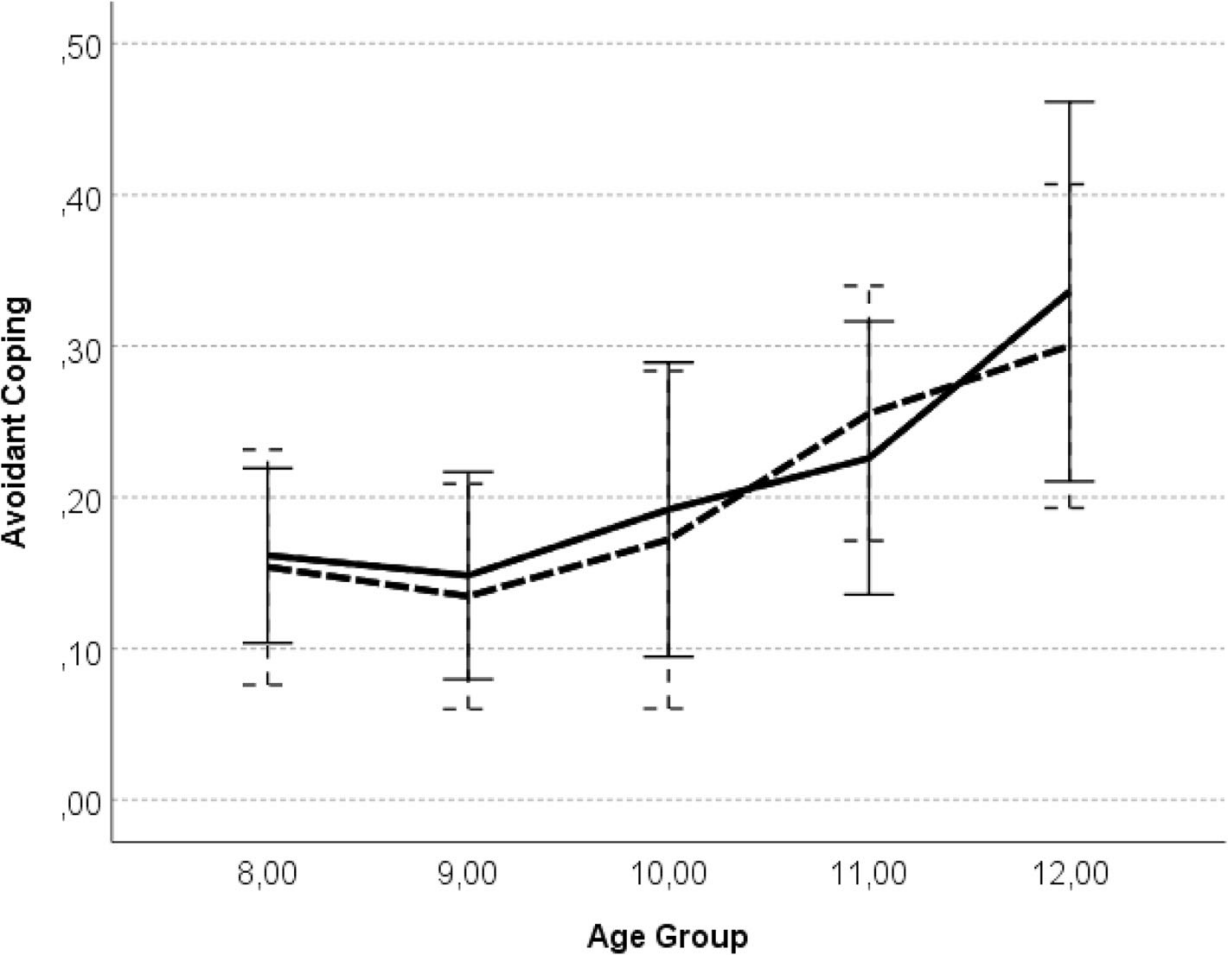
Avoidant coping as a function of child age. *Legend*: error bars represent the standard error; solid line – attachment to mother; dashed line – attachment to father

To examine age (hypothesis 2) and sex (hypothesis 5) differences in avoidant coping strategies, as well as to explore the main effects and possible interactions between child’s child sex, age, as well as emotionality (Hypothesis 6a) and attachment figure, on avoidant coping, a repeated-measures ANOVA with avoidant coping with mothers and fathers as inter-subject variables was conducted. The results showed significant main effect of age group on avoiding coping with both parents, *F* (4, 112) = 3.38, *p* = .01, η_p_^2^ = .14. Tukey’s *post-hoc* tests indicated that: 12-year-olds reported more avoidance with both mother and father than 8-year-olds, *p* = .001, 9-year-olds, *p* = .001, and 10-year-olds, *p* < .01 (see Fig. 2; for mean and standard deviations see Table 2). There were no significant main effects of parent, child sex, emotionality, or the interactions of those variables.

### Age, sex and emotionality differences in preoccupied coping

**Fig. 3:**
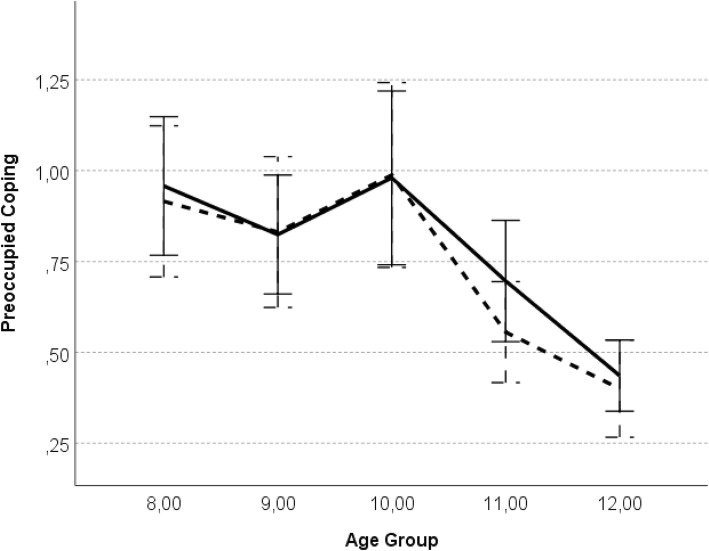
Preoccupied coping as a function of child age. *Legend*: error bars represent the standard error; solid line – attachment to mother; dashed line – attachment to father

To examine age (hypothesis 3) and sex (hypothesis 4) differences in preoccupied coping strategies, as well as to explore the main effects and possible interactions between child’s child sex, age, as well as emotionality (hypothesis 6b) and attachment figure, on preoccupied coping, a repeated-measures ANOVA with preoccupied coping with mothers and fathers as the dependent variables, showed significant main effect of age group, *F* (4, 112) = 7.86, *p* < .000, η_p_^2^ = .22. Tukey’s *post-hoc* tests indicated that 12-year-olds reported significantly less preoccupied coping with both parents than did 8-year-olds, *p* < .001, 9-year-olds, *p* = .001, and 10 year-olds, *p* < .001. Also 11-years-old, reported less preoccupation with parents than did 8-year-olds, *p* < .01, and 10 year-olds, *p* = .01 (see Fig. 3; for mean and standard deviations see Table 2). No significant main effects of parent, child sex, emotionality, or interactions of investigated variables were found.

### Age, sex and emotionality differences in attachment security

**Fig. 4:**
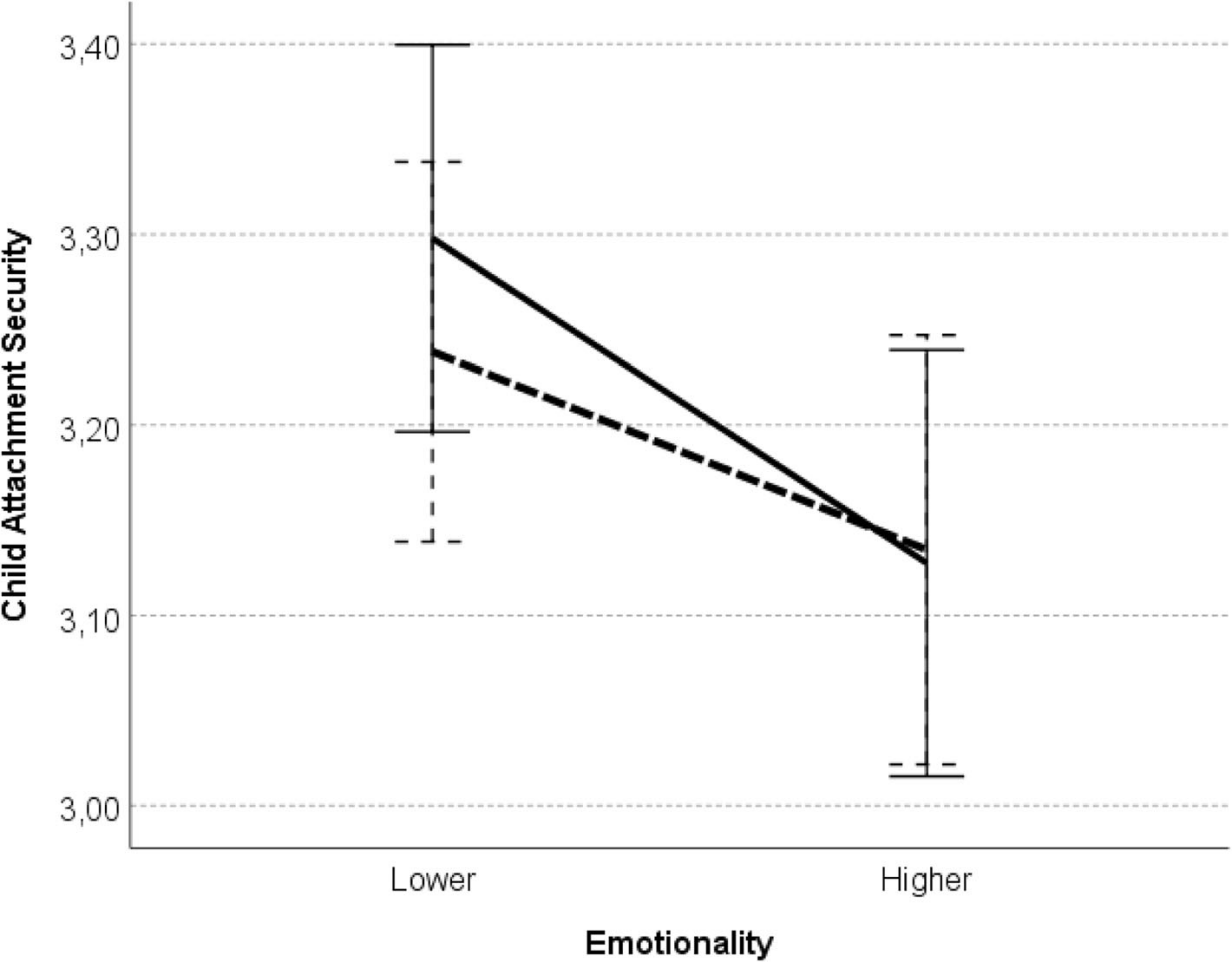
Child attachment security as a function of emotionality. *Legend*: error bars represent the standard error; solid line – attachment to mother; dashed line – attachment to father

To examine an exploratory problem, whether there were main effects as well as interaction effects of attachment figure (i.e. parent sex), child sex, age, as well as emotionality (hypothesis 6c) on perceived attachment security, a repeated measures univariate analysis of variance (ANOVA) was conducted with security with mothers and fathers as the within-subjects factor, and child sex, age and emotionality as the between-subjects factors. Significant main effect of emotionality was found, *F* (1, 112) = 5.52, *p* = .02, η_p_^2^ = .06. Pair-wise comparisons revealed that children with relatively lower emotionality reported higher attachment security with both parents (mother-child security: *M* = 3.30, *SD* = 0.41, father-child security: *M* = 3.24, *SD* = 0.40) than children with relatively higher emotionality (mother-child security: *M* = 3.13, *SD* = 0.45, father-child security: *M* = 3.13, *SD* = 0.46), *p >* .01, Cohen’s *d* = 0.39 (there were no significant differences by parent; see: Fig. 4).

**Fig. 5.**
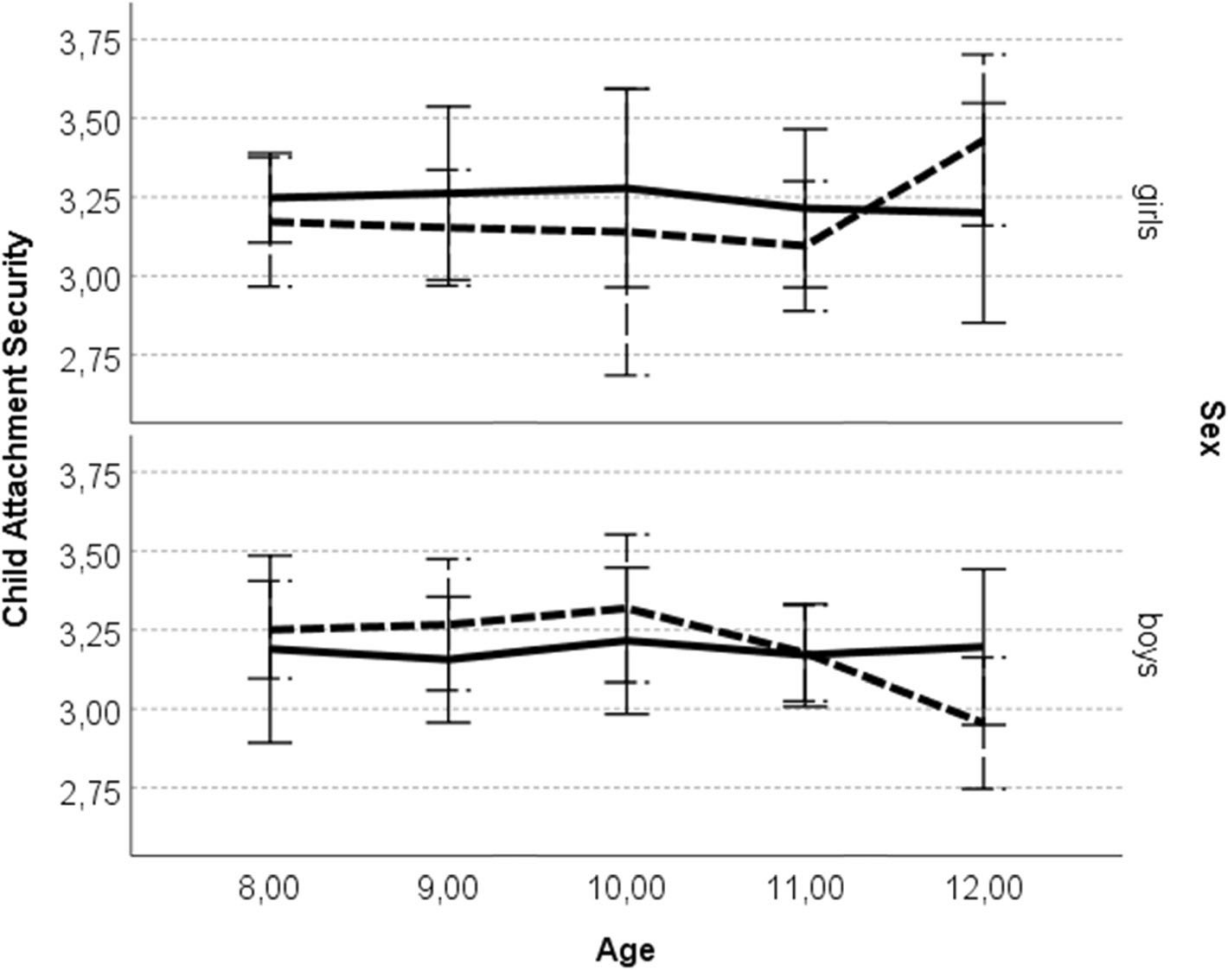
Child attachment security as a function of age and sex. *Legend*: error bars represent the standard error; solid line – attachment to mother; dashed line – attachment to father

Moreover, significant interaction between attachment figure, child’s sex and age was found, *F* (4, 51) = 2.80, *p* = .03, η_p_^2^ = .18. Analysis revealed that among 12-years-old girls attachment security with father (*M* = 3.41, *SD* = 0.42) was higher than security with mother (*M* = 3.17, *SD* = 0.55), *F* (1, 14) = 10.57, *p* = .01, η_p_^2^ = .43 (see: Fig. 5). At the same time, boys did not differ by age with attachment security with parents, *F* (1, 17) = 3.46, *p* = .08, η_p_^2^ = .17. Also, no significant main effects of parent sex, child sex, age or other interactions of investigated variables were found.

## Discussion

The purpose of this study was to examine the developmental trends in children’s perceptions of attachment security with parents as well as preoccupied and avoidant coping strategies with them, as a function of child age, sex, emotionality and attachment figure in middle childhood. The present study is among the first to analyse those developmental trends in the Polish sample drawn from quite a different culture than highly individualistic Western ones, which are over-explored in the attachment literature [37].

Regarding the fact that in Poland the boundaries between collectivistic and individualistic orientations are somewhat blurred, and that Polish mothers are quite overprotective and collectivistic in their socialization goals [46], it was predicted that in general Polish sample children would report more preoccupied than avoidant coping strategies with their parents (hypothesis 1). Results supported that hypothesis for attachment to mothers and fathers, noticeably with a large effect size. Although interpretation of the findings warrants caution because of a relatively quite a small sample size and the potentially shared method variance, the results seem to be consistent with the findings that participants from individualistic countries such as Germany report high levels of avoidant attachment, whereas participants from more collectivist countries - high levels of ambivalent attachment [46]. It is also not surprising, given that in Poland, most of the traditional parenting practices still promote connection to the family and other close relationships, respect, and obedience [51]. Therefore, more research is needed to see whether this effect replicates. Future studies should include a more diverse population (not only the middle-class one) to explore potential within-culture variance in the attachment in middle childhood. As Kroonenberg and Van Ijzendoorn [65] argue, differences in the attachment within a culture might be higher than differences in between cultures since there are no universal childrearing practices for different sub-cultures.

### Age differences in attachment

In line with theoretical predictions [1] and preliminary attachment research in Western countries [1], the current study supports the hypothesis that older children report more avoidant coping strategies with their parents than younger children (hypothesis 2). It might reflect some developmental differences in children’s emotional expression during interactions with parents, along with changes towards greater autonomy and peer-affiliation and the decline in utilization of parents as secure bases and havens with age. They become less reliant on mothers and fathers in the context of the excessive need for assistance and support in stressful or novel situations.

However, in opposite to hypothesis 3, younger children reported more preoccupied coping than the older ones. The observed developmental trend in the Polish sample corresponds to the results from Western studies [e.g., 1]; thus, it seems to reflect rather a culture-universal change in the attachment in middle childhood, related to children’s growing autonomy and self-reliance. Regarding the general higher preoccupied coping in Polish sample, those age differences might also reflect some micro-cultural discrepancies between relatedness-oriented mothers and their autonomy-oriented children, pursuing towards more individualistic goals and orientations in their transition into adolescence [46].

### Sex differences in attachment

A culture-universal link between a child’s sex and attachment insecurity was hypothesized; hence, it was expected that Polish girls would report more preoccupied coping strategies with their parents than boys (hypothesis 4), and boys would report more avoidant coping strategies than girls (hypothesis 5). Although several studies from different cultures have indicated such changes in child relationships with parents in middle childhood [e.g., 16], the present findings failed to find any sex differences in either preoccupied or avoidant coping with either mother or father. This finding contradicts Del Giudice’s [15, 20] claim about the shift towards sex-specific psychosocial reproductive strategies in the transition to adolescence. It is also in contradiction with the socialization-oriented explanations, according to which girls are taught to show affiliate responses, while boys are spurred to react in a more individualistic fashion [21, 51]. One possible explanation of this result is that a child’s sex is not yet a significant factor in middle childhood attachment in Polish culture, while it might gain in significance in adolescence when the social roles of girls and boys become more diverse. It might also be that Polish girls already internalized modern individualistic values and strongly tend to emancipate and to be similarly independent, self-reliant, and autonomous that boys are. However, such a result can be instead due to many other factors, not included in the present study. Future research should include more proximal variables such as parental practices in gender socialization as well as development in cognition and another aspect that might be valid.

### Emotionality and attachment

Another aim of the present study was to check the role of emotionality in middle childhood attachment. Regarding the Bosmans and Kerns [4] claim that in middle childhood biologically determined factors might have more substantial effects on attachment than during infancy and preschool years, it was expected that emotionality, as depicting biologically-grounded negative quality of a child’s emotions and high intensity of emotional reactions, would be positively linked to avoidant (Hypothesis 6a) and preoccupied (hypothesis 6b) coping, and negatively to attachment security (hypothesis 6c) only in older children. However, the results did not support those hypotheses. Instead, they revealed that emotionality does not become more valid for attachment as children age. On the other hand, exploratory analyses revealed that children with relatively lower emotionality reported higher attachment security with both parents than children with relatively higher emotionality. At the same time, there were no significant main effects of emotionality on avoidant and preoccupied coping strategies. Those findings suggest that high degrees of child’s sensitivity to distressing stimuli and a tendency to experience negative emotions might impact a child’s perceptions of caregiver’s availability in middle childhood. It also might make those children interpret parent’s ambiguous behaviour as unsupportive and unresponsive, regardless of the objective meaning of his behaviour [36]. Although the quality of the early child-parent attachment depends at most on the caregiver’s sensitivity and availability to the child’s cues, the concern arises that as children grow older and their cognitive processes develop, those with high negative emotionality might relatively more intensively assimilate biased interpretations of attachment figure secure base and secure haven behaviour in their internal working models. The obtained result also seems to be in line with the studies revealing that children with high levels of difficult temperament are less capable of utilizing their attachment representations to regulate their emotions [35]. Second, our results revealed that emotionality links to reports of general felt-security rather than to attachment preoccupation or avoidance. It might suggest that in middle childhood, temperament might impact the child’s perceptions of the availability and responsivity of the attachment figure in general, rather than impacting the specific degree of the child’s overdependency or denial of the need for the attachment figure. However, longitudinal studies are needed to confirm that hypothesis. Finally, this finding highlights the importance of Younger and colleagues’ [61] claims that attachment security is both conceptually and operationally “something more” than low levels of attachment avoidance and preoccupation, confirming the need to consider multiple measures of attachment in middle childhood, and not only restricting to assessing particular insecurities. Since there is a lack of cross-cultural studies investigating emotionality and attachment in middle childhood, it is quite challenging to discuss obtained results in the context of culture. Future research is needed, including also multiple sources on emotionality assessment. In the present study, it based on mothers’ reports. Regarding that rating of child temperament might differ across the parents [31], it is worth to check, whether similar results would be obtained if fathers assessed the child’s emotionality.

### The role of attachment figure

Another goal of the study was to explore the attachment in middle childhood as a function of the attachment figure, along with possible interaction effects of attachment figure with a child’s characteristics. The analysis revealed that 12-year-old girls reported higher security in attachment with fathers compared to attachment with mothers; however, there were no such differences in boys. That result is in line with previous studies showing increasing paternal availability over time [1], and the other ones reporting lower felt security with mother than with father [24]. Therefore it suggests that for Polish girls, fathers become more open to communication and more responsive in times of need, as compared to mothers by the beginning of adolescence. They might also become somewhat primary attachment figures in the transition to adolescence. However, longitudinal explorations are needed to verify that trend. On the other hand, it is unclear whether fathers do are more akin to serve as secure havens and secure bases for their growing daughters than mothers do, or if other significant factors contribute to this issue. Further research should be conducted to examine more thoroughly the nature of the father-child attachment, including more culture-oriented research.

### Limitations of the study

Although this study adds to the literature by investigating the effects of a child’s characteristics on attachment to mother and father in middle childhood, these findings should also be interpreted with some caution. More specifically, the study’s cross-sectional design does not allow one to investigate the developmental change in the attachment in middle childhood. Therefore, systematic longitudinal studies are necessary to establish how attachment changes in middle childhood and which factors explain the change.

A relatively small sample size and the homogeneity of our sample limits the generalization of these findings only to the middle-class Polish population. It is essential to replicate those results in more ethnically and economically diverse samples. In future studies, single-parent families or adoptive ones should also be included to explore how attachment develops in diverse family structures, and whether there are some interactions between cultural values and family structures.

There are also some validity concerns around self-report measures such as difficulties in conscious access to internal working models, the risk for response bias and social desirability, or shared method bias [4]. Hence some alternative tools should be used in future studies to corroborate the significance of the presented findings. Analysing children’s spontaneous narratives might give some more insights into cultural differences between children’s perceptions of attachment and their mental representations of the *self* in Poland and other cultures (cf. [66]). Several recent studies (e.g., [58]) reveal that self-reports on attachment correlate with narrative measures of attachment representations in middle childhood. Moreover, measures we used might be culturally biased, especially towards the Western way of thinking about attachment [7], −as some authors call into question the cultural universality of such construct as a secure base, individuation, and exploration (see *Background*). It is also worth to note that in the present study, a child’s emotionality was assessed through a self-report completed by mothers. More indirect, physiological measures of the child’s emotionality would add further information to this area of study.

Finally, as Tamis-LeMonda [38] argues, cultural values and family developmental goals may dynamically coexist in different constellations as conflicting, additive, or functionally dependent. Therefore, future studies would be more informative if dominant cultural patterns, along with individual cultural orientations, were assessed directly and then examined with individual differences and developmental changes in the attachment in different cultures.

## Conclusions

The present study is the first one to analyse developmental trends in the attachment in Polish sample, drawn from quite a different culture than highly individualistic Western ones, which are over-explored in the attachment literature [37]. The current study extends previous work on attachment in middle childhood not only by examining developmental trends in Polish sample but also by including the attachment to fathers and testing a possible, increasing role of temperament on attachment to both parents after infancy and preschool years. The findings broaden our knowledge about individual differences in attachment during middle childhood, an area of research that has been neglected relative to research on other periods of the life span. Some of the results replicated prior studies conducted in Western cultures (i.e., with older children being less preoccupied and more avoidant in their coping strategies with parents, and older girls reporting higher felt-security with their fathers than with mothers). However, some novel findings were also found (i.e., no links between child’s sex and preoccupied and avoidant coping; higher rates of preoccupied coping than the avoidant one in the general sample; higher attachment security with both parents in children with relatively lower emotionality as compared to children with the relatively higher one. At the current stage of the research, it is difficult to say whether those results might also be generalized to children coming from other Eastern European countries. Regarding the fact that among other eastern countries, Poland is overcoming the most advanced socio-political change, it seems possible that there is some specificity in the Polish sample. However, any inferences should be made with caution until more replication studies are conducted.

## Data Availability

The datasets used and analysed during the current study are available from the corresponding author on reasonable request.

## Abbreviations

CSQ: Coping Strategies Questionnaire
EAS-C: Emotionality, Activity, and Sociability Survey for Children
KSS: Kern’s Security Scale
SES: socioeconomic status
